# PARP Inhibition potentiates boron neutron capture therapy in chemoresistant glioblastoma via DNA repair disruption

**DOI:** 10.1007/s11604-025-01929-9

**Published:** 2025-12-24

**Authors:** Ting-Yu Zhou, Zih-Yin Lai, Tzu-Jung Hsu, Zheng-Shun Xu, Yi-Wei Chen, Fong-In Chou, Yung-Jen Chuang

**Affiliations:** 1 School of Medicine, National Tsing Hua University, Hsinchu, Taiwan, ROC; 2 Institute of Bioinformatics and Structural Biology, National Tsing Hua University, Hsinchu, Taiwan, ROC; 3 Nuclear Science and Technology Development Center, National Tsing Hua University, Hsinchu, Taiwan, ROC; 4 Department of Heavy Particles and Radiation Oncology, Taipei Veterans General Hospital, Taipei, Taiwan, ROC

**Keywords:** BNCT, PARP inhibitor, GBM, Chemoresistant, DNA damage and repair

## Abstract

**Background:**

Glioblastoma (GBM) is the most aggressive primary brain tumor, with poor responsiveness to existing therapies and no established second-line treatment for recurrence. Boron neutron capture therapy (BNCT) has emerged as a promising modality that delivers selective cytotoxicity to recurrent GBM, yet its efficacy is constrained by tumor-intrinsic DNA repair mechanisms. Targeting DNA repair pathways may therefore represent a rational strategy to potentiate BNCT and improve outcomes in TMZ-resistant GBM.

**Methods:**

We investigated whether combining BPA-mediated BNCT with the PARP inhibitor olaparib enhances efficacy in temozolomide (TMZ)-resistant U-87 TR and parental U-87 MG GBM cells. Clonogenic assays were used to quantify cytotoxicity, while mechanistic studies evaluated DNA damage, cell cycle arrest, and apoptosis.

**Results:**

Olaparib significantly sensitized GBM cells to BNCT, reducing survival to 40.7 ± 8.1% in U-87 MG and 24.2 ± 8.3% in U-87 TR cells, with radiation enhancement ratios of 1.53 and 1.95, respectively. In U-87 TR cells, the combination treatment induced persistent γH2AX foci, sustained G_2_/M arrest, and suppressed BNCT-driven upregulation of BRCA1 and RAD51, indicating impaired HR repair. Apoptosis was markedly increased in both cell lines, proceeding through PUMA–BAX activation in U-87 MG and via PUMA-independent pathways in U-87 TR.

**Conclusions:**

PARP inhibition (Olaparib) enhances BNCT-induced DNA damage, disrupts HR repair, and induces apoptosis through distinct mechanisms in sensitive and resistant GBM cells. These findings provide mechanistic evidence for BNCT–PARP inhibitor combinations as a strategy to overcome therapeutic resistance and merit further translational and clinical investigation in TMZ-resistant GBM.

**Graphical abstract:**

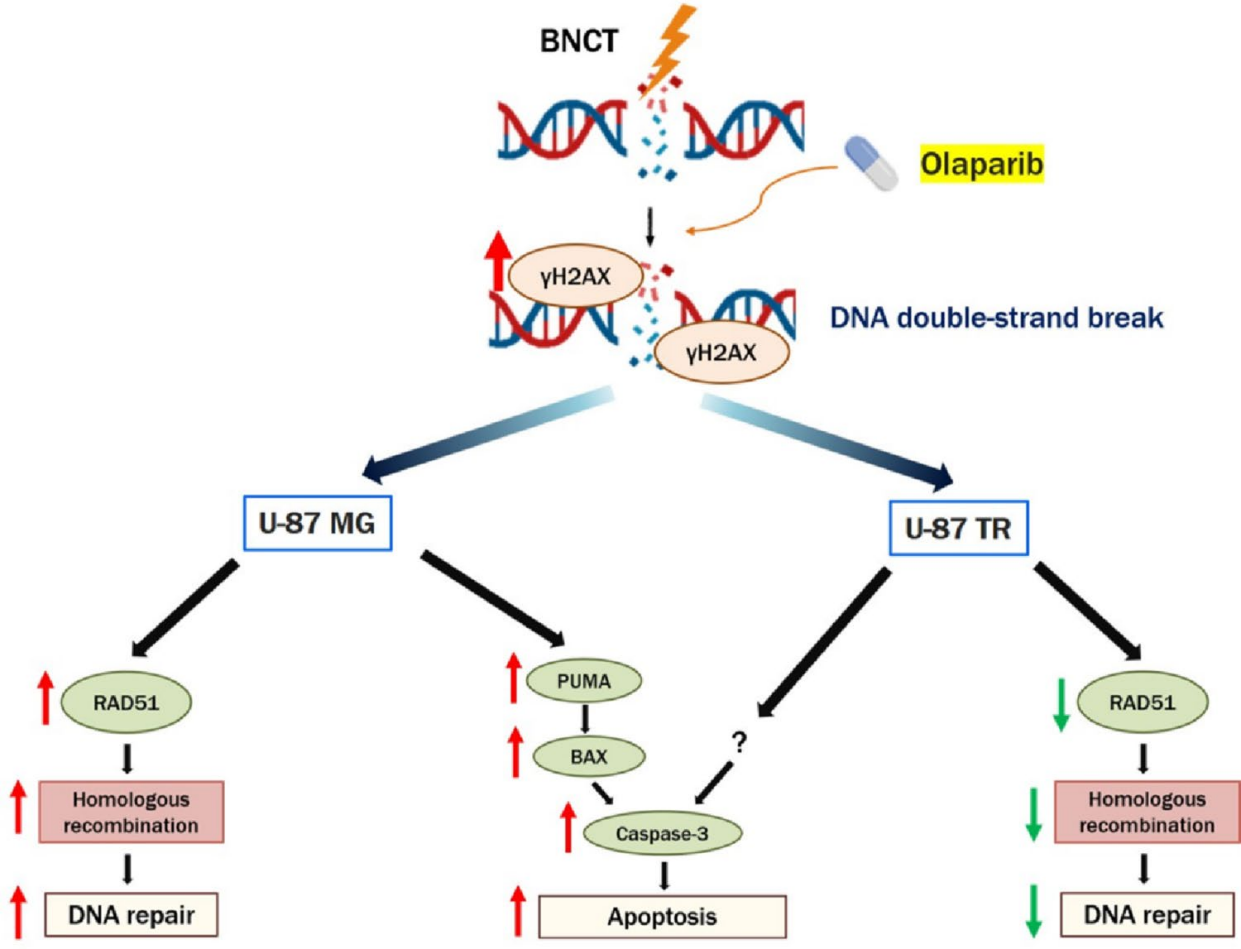

**Supplementary Information:**

The online version contains supplementary material available at 10.1007/s11604-025-01929-9.

## Introduction

Glioblastoma (GBM) is the most aggressive and prevalent primary malignant brain tumor in adults, with a median survival of 15 months and a 5-year survival rate of around 5% [1]. Its poor prognosis stems from rapid proliferation, diffuse infiltration, and pronounced inter- and intra-tumoral heterogeneity [1]. Standard treatment, including maximal safe surgical resection followed by concurrent radiotherapy and temozolomide (TMZ) chemotherapy, has been established by the landmark Stupp protocol [2]. This multimodal approach provides only temporary disease control; most GBM patients develop recurrent disease within months [1, 3]. Recurrent GBM is difficult to treat because tumor cells evade therapy through stem-like subpopulations, extensive genetic heterogeneity, and protection by the blood–brain barrier (BBB). Recurrence is also driven by enhanced DNA repair capacity and defective apoptotic signaling [4–6]. These resistance mechanisms severely limit durable clinical responses, underscoring the urgent need for innovative therapeutic strategies to overcome them.

Boron neutron capture therapy (BNCT) is a targeted radiation modality that selectively destroys boron-loaded tumor cells through high-linear energy transfer particles generated by a nuclear reaction between boron-10 (^10^B) and thermal neutrons [7]. This approach could spare the surrounding healthy tissue and has shown clinical promise in treating recurrent GBM [8–11]. In Taiwan, the Tsinghua open pool reactor (THOR) has been central to BNCT development, with clinical evidence suggesting it can delay brain tumor progression and preserve quality of life [9]. However, tumor recurrence remains common after BNCT, highlighting the need for rational combination strategies to improve tumor control, particularly in therapy-resistant disease [12–14].

Resistance to cytotoxic therapy in GBM is often linked to enhanced DNA damage response (DDR) activity, primarily upregulated homologous recombination (HR) repair of double-strand breaks (DSBs) [15, 16]. BNCT induces complex and clustered DNA lesions that are predominantly resolved through HR [17, 18]. In our prior analysis, TMZ-resistant U-87 TR cells displayed elevated HR activity following BNCT, which supported their survival and contributed to treatment resistance (Supplementary Figs. 1 and 2). These findings provide a clear rationale for combining BNCT with agents blocking tumor cell DNA repair.

Poly(ADP-ribose) polymerase (PARP) plays a key role in the base excision repair (BER) pathway and facilitates early DSB recognition and HR activation [19–22]. PARP inhibitors (PARPi) exploit synthetic lethality in HR-deficient tumors and have demonstrated clinical efficacy in BRCA-mutated cancers [23–25]. Among available PARP inhibitors, olaparib has been prioritized due to its favorable pharmacokinetic properties and established clinical safety profile in patients with recurrent malignancies [26]. Preclinical evidence indicates that olaparib radiosensitizes tumor cells by enhancing DNA damage and disrupting repair mechanisms [27, 28], supporting its potential to potentiate BNCT-induced cytotoxicity in resistant GBM.

The mechanistic rationale for combining BNCT with PARP inhibition lies in delivering a synergistic disruption of DNA repair, since BNCT imposes a high burden of complex DSBs on tumor cells [22, 29]. Specifically, BNCT induces extensive DNA damage, particularly DSBs, and burdens the tumor cell’s repair machinery [17, 19, 30]. Meanwhile, concurrent inhibition of PARP impairs SSB repair, leading to replication-associated DSB accumulation and genomic instability [22, 29]. This dual assault can overwhelm the DDR network, inducing cell death even in DNA repair–proficient, chemoresistant GBM. Preclinical studies have confirmed the radiosensitizing effects of PARPi and their synthetic lethal interaction with radiation [27, 31, 32]. Because both BNCT and olaparib are clinically relevant modalities, this combination has direct translational potential for TMZ-resistant GBM.

In this study, we investigate whether combining olaparib with BPA-mediated BNCT can overcome DNA repair–mediated resistance in GBM. We hypothesize that this strategy enhances BNCT efficacy by amplifying DNA damage, disrupting DNA repair pathways, and promoting apoptosis in resistant cells. Specifically, we aimed to determine whether PARP inhibition potentiates BNCT-induced cytotoxicity in temozolomide-resistant GBM and to elucidate the molecular mechanisms underlying this interaction.

## Materials and methods

### Cell lines

The human glioblastoma (GBM) cell line U-87 MG was obtained from the Bioresource Collection and Research Center (BCRC No. 60360; Hsinchu, Taiwan). Cells were cultured in Minimum Essential Medium α (MEM-α; HyClone, USA) supplemented with 10% heat-inactivated fetal bovine serum (FBS), 100 U/mL penicillin, and 100 µg/mL streptomycin. Cell cultures were maintained at 37 °C in a humidified incubator with 5% CO_2_. The temozolomide (TMZ)-resistant GBM cell line, U-87 TR, was established previously in our laboratory through stepwise exposure to increasing concentrations of TMZ (Supplementary Fig. 3). U-87 TR cells were cultured under the same conditions as U-87 MG cells to ensure consistency in experimental comparisons.

### Drug preparation and treatment protocols

l-(4-^10^Boronophenyl) alanine fructose injection (L-BPA; Taiwan Biotech Co., Ltd.) was provided by Professor Fong-In Chou (Institute of Nuclear Engineering and Science, National Tsing Hua University, Hsinchu, Taiwan). A stock solution of L-BPA was prepared at a concentration of 1272 µg ^10^B/mL and stored at 4 °C. Before use, the stock solution was sterilized by filtration through a 0.22 μm membrane filter.

### Neutron irradiation procedure

For neutron irradiation experiments, 2 × 10^5^ cells were seeded into 6-well plates and cultured for 48 h. At 24 h post-seeding, cells assigned to the olaparib and combination treatment groups were treated with 20 µM olaparib. Cells of relevant groups were incubated with ^10^B-BPA for 30 min before neutron exposure. Following incubation, the culture medium was aspirated, and the culture plates were transported to the Tsinghua open-pool reactor (THOR; Hsinchu, Taiwan) for neutron irradiation. The physical dose rate and neutron flux of THOR are summarized in Supplementary Table 1.

BNCT was performed under fixed reactor beam conditions with standardized irradiation parameters. The absorbed dose was expressed as the physical neutron dose (Gy), calculated from neutron fluence, and no boron-dependent biological dose correction (Gy-Eq matching) was applied.

### Cell viability assay and IC_50_ determination

U-87 MG and U-87 TR cells were seeded in 96-well plates and incubated for 24 h for cell attachment. Thereafter, cells were treated with a range of olaparib concentrations for 72 h. Cell viability was assessed using the Cell Counting Kit-8 (CCK-8; Dojindo, Kumamoto, Japan) according to the kit manufacturer’s protocol. Following the addition of the CCK-8 reagent, cells were incubated for 1 h, and absorbance was measured at 450 nm using a microplate reader. The half-maximal inhibitory concentration (IC_50_) was calculated by nonlinear regression analysis using GraphPad Prism 9 software (GraphPad Software, San Diego, CA, USA).

### Colony formation assay

Following BNCT irradiation, U-87 MG and U-87 TR cells were reseeded into 6-well plates at a density of 600 cells per well and incubated under standard culture conditions until visible colonies formed. Colonies were then fixed with 95% methanol (Merck, Darmstadt, Germany) and stained with 0.1% crystal violet (Sigma-Aldrich, Steinheim, Germany). Excess stains were removed by rinsing with tap water, and the plates were subsequently air-dried at room temperature. The survival fraction (SF) was calculated as the ratio of the number of colonies in the irradiated groups to that in the corresponding non-irradiated control groups.

### Immunocytochemistry (ICC) for DNA damage markers

Immediately following neutron irradiation, 3500 U-87 MG and U-87 TR cells were seeded onto sterile glass coverslips. At 10 and 24 h post-BNCT irradiation, cells were fixed with 4% paraformaldehyde (PFA; Sigma-Aldrich, Steinheim, Germany), permeabilized with 0.1% Triton X-100, and incubated overnight at 4 °C with 0.25 µL (1:400 dilution) of anti-γH2AX antibody (Ser139; #9718, Cell Signaling Technology, Danvers, MA, USA). Then, the secondary antibody, anti-rabbit IgG-DyLight 488 (0.25 µL, 1:400; Jackson ImmunoResearch, West Grove, PA, USA) was added and incubated for 2 h at 37 °C. Nuclei were counterstained with Hoechst 33342 (1:200; Invitrogen, Carlsbad, CA, USA) for 15 min at 37 °C. The coverslips were mounted using antifade mounting medium (Invitrogen, Eugene, OR, USA). Immunofluorescence images were acquired using an LSM800 confocal microscope (Zeiss, Oberkochen, Germany).

### Cell cycle analysis via flow cytometry

At 10, 24, and 48 h post-BNCT irradiation, cells were harvested and fixed in 80% ethanol. DNA staining was performed per the manufacturer’s protocol (BD Biosciences, San Diego, CA, USA). Briefly, fixed cells were washed with PBS, followed by BD Pharmingen™ Stain Buffer (554656; BD Biosciences), and subsequently stained with BD Pharmingen™ 7-amino-actinomycin D (7-AAD) staining solution (559925; BD Biosciences) for 15 min at room temperature in the dark. Cell suspensions were filtered through a 35 μm nylon mesh cell strainer (Falcon, Reynosa, Tamaulipas, Mexico) to eliminate clumps and analyzed using a CytoFLEX flow cytometer (Beckman Coulter, Indianapolis, IN, USA). Cell cycle distribution was determined based on DNA content profiles.

### Western blot assay

U-87 MG and U-87 TR cells were harvested at 10, 24, and 48 h post-irradiation. Total protein was extracted using RIPA lysis buffer (Roche, Indianapolis, IN, USA) and quantified using the Bicinchoninic Acid (BCA) Protein Assay Kit (Thermo Scientific, Rockford, IL, USA). Equal amounts of protein (30 µg per sample) were separated by SDS-PAGE and subsequently transferred onto PVDF membranes (Millipore, Darmstadt, Germany). After blocking with 3% BSA, membranes were incubated with primary antibodies overnight at 4 °C, followed by HRP-conjugated secondary antibodies (GE Healthcare, Buckinghamshire, UK; GeneTex, Hsinchu City, Taiwan). Protein bands were visualized using the ImageQuant LAS 4000 mini system (GE Healthcare, Chicago, IL, USA), and densitometry was performed with ImageJ software (NIH, Bethesda, MD, USA). A detailed list of the primary and secondary antibodies used is provided in Supplementary Table 2.

### Apoptosis detection by Caspase-3 activity

Apoptosis was assessed using the PE Active Caspase-3 Apoptosis Assay Kit (BD Biosciences, San Diego, CA, USA), following the manufacturer’s protocol. U-87 MG and U-87 TR cells were harvested 24 and 48 h after BNCT irradiation. Cells were washed with PBS and incubated with a phycoerythrin (PE)-conjugated rabbit anti-active caspase-3 antibody for intracellular staining. Following staining, cell suspensions were passed through a 35 μm nylon mesh cell strainer (Falcon, Reynosa, Tamaulipas, Mexico) to remove aggregates. Flow cytometric analysis was performed using a CytoFLEX flow cytometer (Beckman Coulter, Indianapolis, IN, USA), and data were acquired and analyzed using CytExpert software (Beckman Coulter).

### Statistical analysis

All experimental data are presented as the mean ± standard deviation (SD) from three independent experiments. Statistical differences between two groups were evaluated using unpaired, two-tailed Student’s *t*-tests. Pairwise comparisons were performed only between predefined experimental groups based on specific hypotheses. Therefore, no adjustment for multiple testing was applied. A *p* value of < 0.05 was considered statistically significant. All analyses were performed using GraphPad Prism 9 software (GraphPad Software, San Diego, CA, USA).

## Results

### Olaparib functions as a potential radiosensitizer in BNCT

**Fig. 1:**
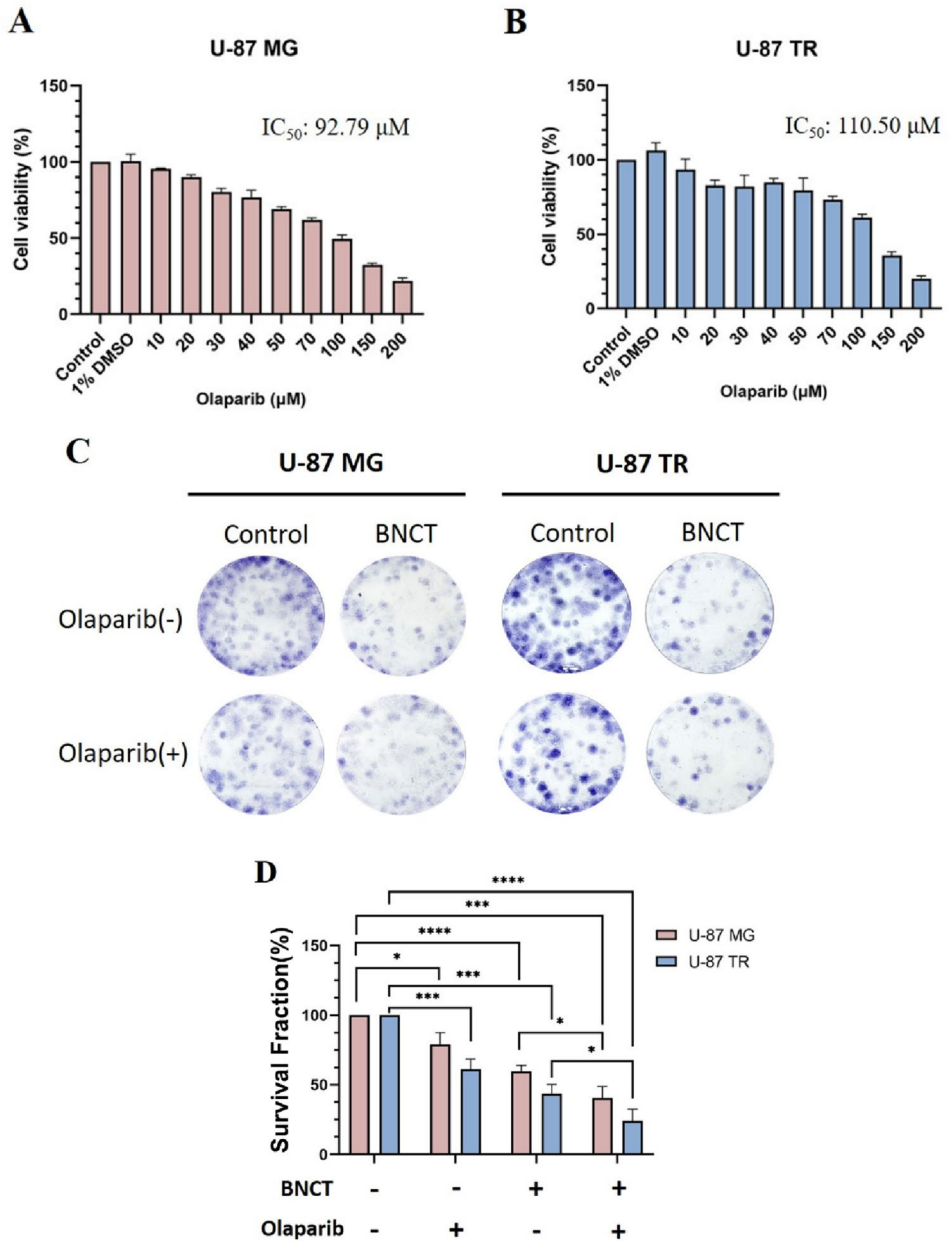
Cell Viability and Clonogenic Survival After Olaparib and BNCT Treatment. **A** and **B** Dose–response analysis of the olaparib in U-87 MG and temozolomide-resistant U-87 TR glioblastoma cells, assessed by the CCK-8 assay. **C** Representative colony formation images of cells treated with olaparib (20 µM), BNCT (3 Gy), or the combination. The control group received no treatment. Colonies were fixed and stained with crystal violet. **D** Quantification of surviving fractions based on colony-forming ability. Combination treatment significantly reduced clonogenic survival in both cell lines compared with BNCT alone. Note: CCK‑8 **A** and **B** reflects 72‑h short-term metabolic activity, whereas clonogenic survival **C** and **D** measures long‑term reproductive capacity; values are not directly comparable. Data are presented as mean ± standard deviation (SD) from three independent experiments. Statistical significance was assessed by an unpaired, two-tailed Student’s *t*-test with multiple comparisons (*p < 0.05, ***p < 0.001, ****p < 0.0001)

To determine the appropriate concentration of olaparib for treating GBM cells, we first evaluated its cytotoxic effects on U-87 MG and U-87 TR cells using the CCK-8 assay. The calculated IC_50_ values were 92.79 µM for U-87 MG and 110.50 µM for U-87 TR cells (Fig. 1A, B), indicating similar sensitivity profiles. A concentration-dependent decrease in cell viability was observed in both lines. The IC_10_ values for both cell types were approximately 20 µM, which aligns with previous reports demonstrating radiosensitization at sub-cytotoxic doses [33]. Therefore, 20 µM olaparib was selected for subsequent experiments.

To investigate whether olaparib could enhance the cytotoxic effects of BPA-mediated BNCT, U-87 MG and U-87 TR cells were treated with 3 Gy BNCT alone or in combination with 20 µM olaparib. The 3 Gy irradiation dose was selected based on our BNCT dose–response analysis (Supplementary Fig. 4), which showed that this dose produced a measurable cytotoxic effect in both cell lines while maintaining sufficient surviving fractions to evaluate radiosensitization. A colony formation assay was performed to assess long-term cell viability following treatments (Fig. 1C). As shown in Fig. 1D, co-treatment significantly reduced the survival fraction to 40.67% ± 8.10% in U-87 MG and 24.20% ± 8.29% in U-87 TR cells, compared to 59.84% ± 4.07% and 43.64% ± 6.50%, respectively, in the BNCT alone groups.

The radiation enhancement ratio (RER) was calculated to quantify the radiosensitizing effect further. An RER greater than 1 indicates that olaparib enhances radiation-induced cytotoxicity, with higher values reflecting greater sensitization potential [34]. Following 3 Gy BNCT irradiation, RER values exceeded 1 in both cell lines: 1.531 ± 0.450 for U-87 MG and 1.952 ± 0.774 for U-87 TR cells (Supplementary Table 3), showing a stronger radiosensitizing trend in chemoresistant U-87 TR cells, although the difference was not statistically significant (*p* > 0.05). These findings support the potential of olaparib as a BNCT radiosensitizer, especially for treating TMZ-resistant glioblastoma.

### Prolonged DNA damage response following olaparib and BNCT in U-87 TR cells

**Fig. 2:**
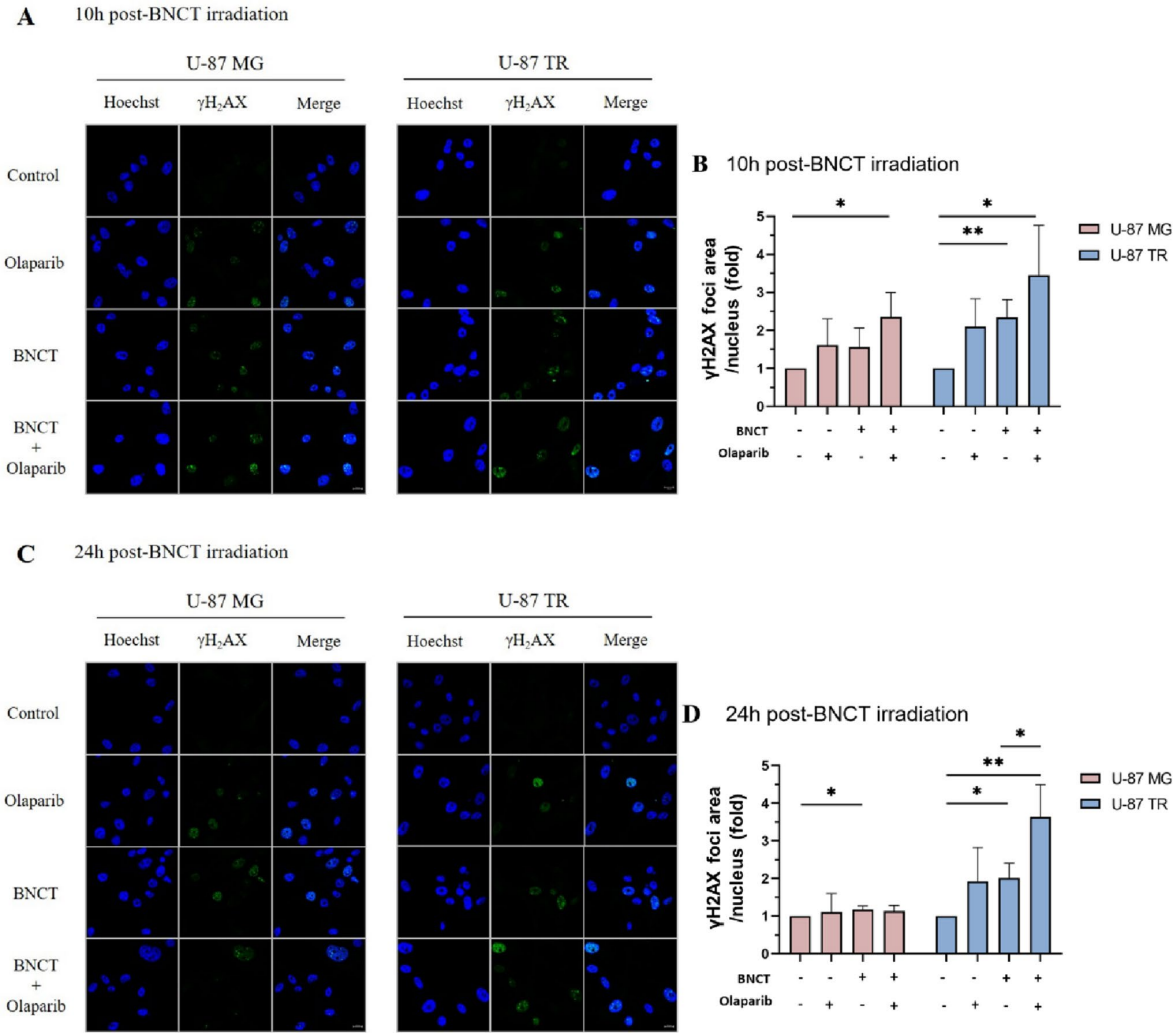
DNA damage (γH2AX foci formation) following combined treatment. **A** and **C** Representative ICC images of γH2AX foci (green) and nuclear (Hoechst, blue) in U-87 MG and TMZ-resistant U-87 TR cells at 10 h (**A**) and 24 h (**C**) post-BNCT irradiation. Cells were treated with either olaparib (20 µM), BNCT (3 Gy), or the combination. The control group represents untreated cells (no treatment). All images were taken at the same magnification; scale bars: 10 μm. **B** and **D** Quantification of γH2AX foci area relative to nuclear area. Data are shown as mean ± SD from three independent experiments. Statistical significance was assessed using an unpaired, two-tailed Student’s *t*-test with multiple comparisons (*p < 0.05, **p < 0.01)

BNCT exerts its cytotoxic effects primarily by inducing clustered DNA damage, particularly DSBs, which are the most deleterious among these lesions and a key determinant of cell death and genomic instability [19, 30]. To determine whether olaparib could potentiate BNCT-induced DSBs, we examined γH2AX foci formation, a well-established biomarker of DSBs, in U-87 MG and U-87 TR cells (Fig. 2).

At 10 h post-irradiation (Fig. 2A, quantification shown in Fig. 2B), BNCT increased γH2AX foci levels in U-87 MG cells (1.565 ± 0.494-fold), while a more substantial elevation was observed in U-87 TR cells (2.338 ± 0.476-fold). Combination with olaparib further elevated γH2AX foci formation in both cell lines, with U-87 MG reaching 2.352 ± 0.644-fold and U-87 TR increasing to 3.452 ± 1.310-fold. Although the relative enhancement over BNCT alone was similar (approximately 1.5-fold) in both cell lines, the absolute γH2AX level was higher in U-87 TR cells.

At 24 h post-irradiation (Fig. 2C, quantification shown in Fig. 2D), γH2AX foci levels in U-87 MG cells declined to near-baseline levels. In contrast, U-87 TR cells maintained elevated levels of γH2AX foci after BNCT treatment (2.016 ± 0.392-fold), and this persistence was even more pronounced in the combination group (3.636 ± 0.860-fold).

These findings indicate that olaparib enhances and prolongs BNCT-induced DNA damage in U-87 TR cells, as evidenced by sustained γH2AX foci formation. This observation suggests that olaparib may potentiate the therapeutic efficacy of BNCT in chemoresistant GBM cells by enhancing and sustaining DNA damage responses, although the precise molecular mechanisms require further study.

### Olaparib augments and prolongs G_2_/M phase arrest induced by BNCT in U-87 TR cells

**Fig. 3:**
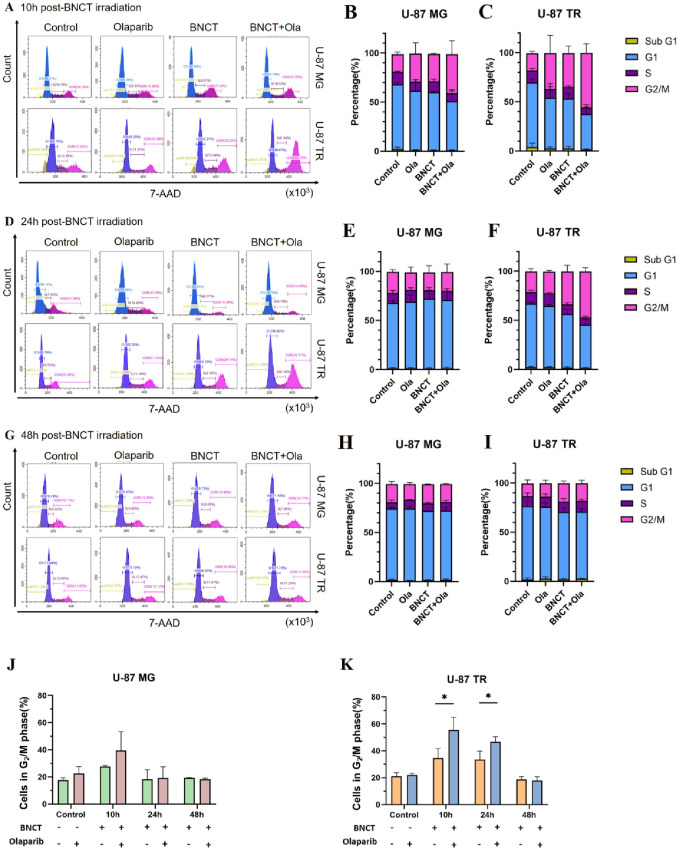
G_2_/M cell cycle arrest induced by treatment. **A**, **D** and **G** Representative histograms of cell cycle distribution in U-87 MG and U-87 TR cells at 10 h (**A**), 24 h (**D**), and 48 h (**G**) post-BNCT irradiation, analyzed by 7-AAD DNA staining. Cells were treated with olaparib (20 µM), BNCT (3 Gy), or their combination. The control group represents untreated cells (no treatment). **B**, **C**, **E**, **F**, **H** and **I** Stacked bar plots showing cell cycle phase distribution. **J** and **K** Quantification of cells in G_2_/M-phase populations over time. Data are shown as mean ± SD from three independent experiments. Statistical analysis was performed using an unpaired, two-tailed Student’s *t*-test (*p < 0.05)

Following radiation-induced DNA damage, cells activate checkpoint mechanisms to regulate cell cycle progression and maintain genomic stability [35]. The G_2_/M checkpoint prevents mitotic entry until DNA lesions are properly repaired [35, 36]. PARP inhibitors such as olaparib are known to interfere with HR and may prolong G_2_/M arrest when DNA repair is incomplete [37]. To examine whether olaparib affects cell cycle dynamics following BNCT irradiation, 7-AAD staining was performed (Fig. 3).

At 10 h post-irradiation (Fig. 3A, quantification shown in Fig. 3B and C), BNCT treatment modestly increased the G_2_/M-phase population in U-87 TR cells (34.530% ± 7.188%) compared to U-87 MG cells (27.620% ± 0.823%). Co-treatment with olaparib resulted in a more pronounced G_2_/M arrest in U-87 TR cells to 55.567% ± 9.346%, while the increase in U-87 MG cells (39.527% ± 13.888%) was not statistically significant. These findings suggest that olaparib promotes early engagement of the G_2_/M checkpoint, particularly in the chemoresistant U-87 TR cell line.

At 24 h post-irradiation (Fig. 3D, quantification shown in Fig. 3E and F), G_2_/M arrest was no longer evident in U-87 MG, but remained elevated in U-87 TR cells, particularly in the combination group (46.793% ± 3.755%) compared to the BNCT monotherapy group (33.497% ± 6.198%). By 48 h post-irradiation (Fig. 3G, quantification shown in Fig. 3H and I), G_2_/M arrest was no longer detectable in either cell line across all treatment groups. A summary of G_2_/M-phase distribution across all timepoints is provided in Fig. 3J and K for U-87 MG and U-87 TR cells, respectively.

These findings demonstrate that olaparib enhances and prolongs BNCT-induced G_2_/M phase arrest in U-87 TR cells. This observation supports the anticipated role of olaparib in enhancing DNA damage response activation and delaying cell cycle progression in chemoresistant GBM. However, the precise molecular mechanisms underlying these effects remain to be fully elucidated.

### Modulation of CDK1 checkpoint signaling by combined treatment

**Fig. 4:**
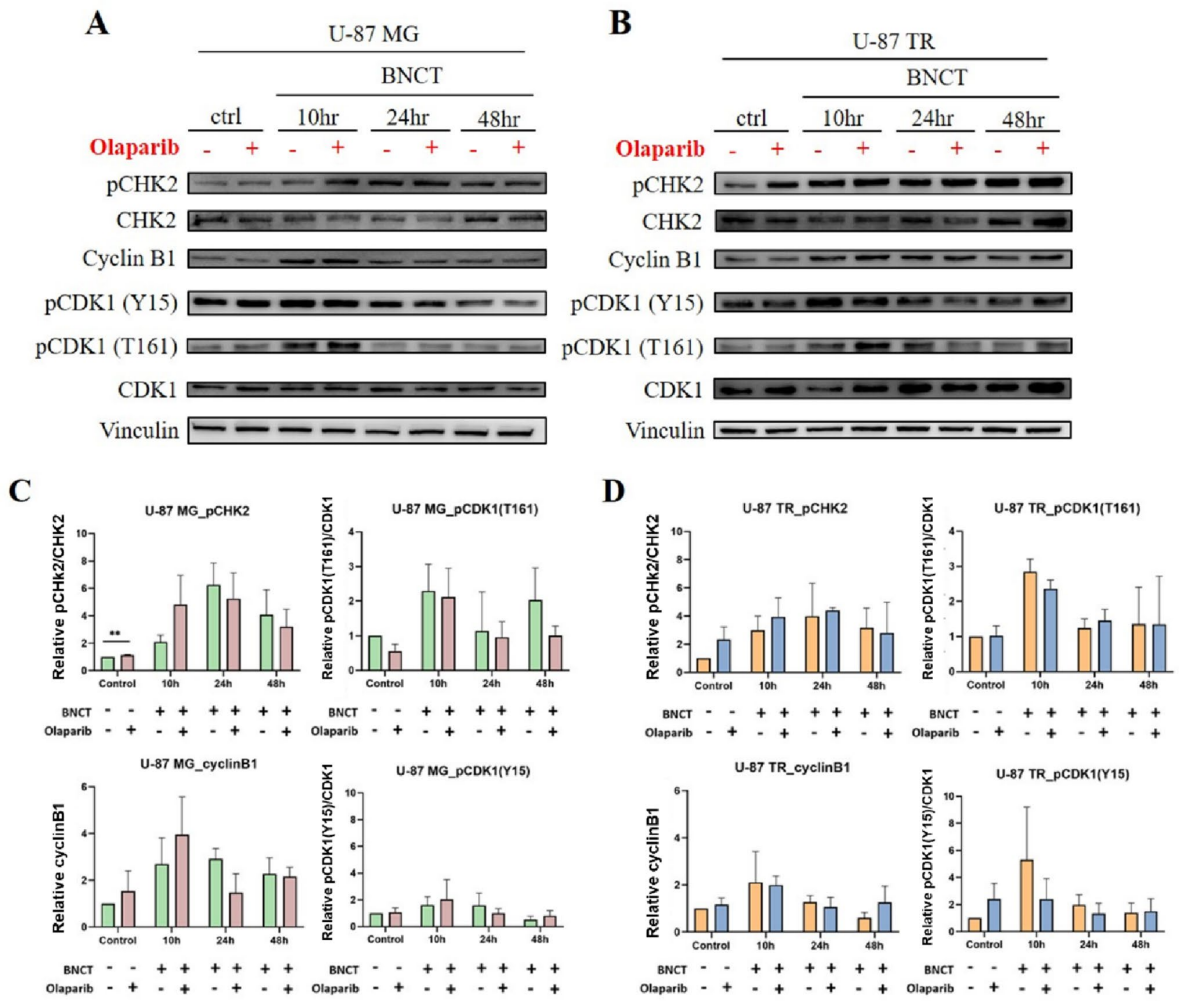
CDK1 checkpoint signaling in response to combined treatment. Representative Western blot analyses of G_2_/M checkpoint regulatory proteins in U-87 MG (**A**) and TMZ-resistant U-87 TR (**B**) cells at 10, 24, and 48 h post-treatment. Vinculin served as a loading control. **C** and **D** Densitometric quantification of the expression levels normalized to total protein or vinculin. Data are shown as mean ± SD from three independent experiments. Statistical analysis was performed using an unpaired, two-tailed Student’s *t*-test (**p < 0.01)

To determine whether the observed G_2_/M arrest was mediated by checkpoint activation, we examined key regulatory proteins, including CHK2, Cyclin B1, and CDK1 at its inhibitory (Y15) and activating (T161) phosphorylation sites (Fig. 4). CHK2 phosphorylation indicates checkpoint activation, while CDK1-Cyclin B1 complexes regulate mitotic entry. Phosphorylation of CDK1 at Y15 inhibits cell cycle progression, whereas phosphorylation of T161 promotes mitotic entry [38].

In U-87 MG cells, BNCT monotherapy markedly increased pCHK2 expression at all time points, with similar patterns observed in the combination group. Cyclin B1 levels progressively increased after BNCT treatment but declined rapidly with combination treatment, possibly reflecting early checkpoint engagement or transition into apoptosis. Phosphorylation of CDK1 at Y15 (pCDK1-Y15) increased moderately following BNCT treatment and peaked at 10 h in the combination group, but returned to near-baseline by 24 h, possibly due to checkpoint adaptation or repair resolution.

In contrast, U-87 TR cells showed sustained pCHK2 and pCDK1-Y15 expression, especially after combination treatment, indicating prolonged checkpoint activation and G_2_/M arrest. Cyclin B1 levels also declined rapidly in U-87 TR cells, further supporting a shift toward sustained arrest or apoptotic signaling. Interestingly, the phosphorylation of CDK1 at T161 increased across both cell lines and treatments. These results suggest that despite activation signals promoting mitotic entry, the concurrent and sustained Y15 phosphorylation overrides this activation, effectively locking cells in the G₂/M phase.

Taken together, these results support a model in which combined olaparib and BNCT treatment alters the phosphorylation dynamics of CDK1, particularly through sustained pCDK1-Y15 expression in U-87 TR cells, thereby reinforcing G_2_/M checkpoint arrest. This extended arrest reflects an active DNA damage response, which may influence cell fate, depending on whether repair is achieved or apoptotic pathways are activated.

### Disruption of homologous recombination (HR) pathway proteins by olaparib in U-87 TR cells

**Fig. 5:**
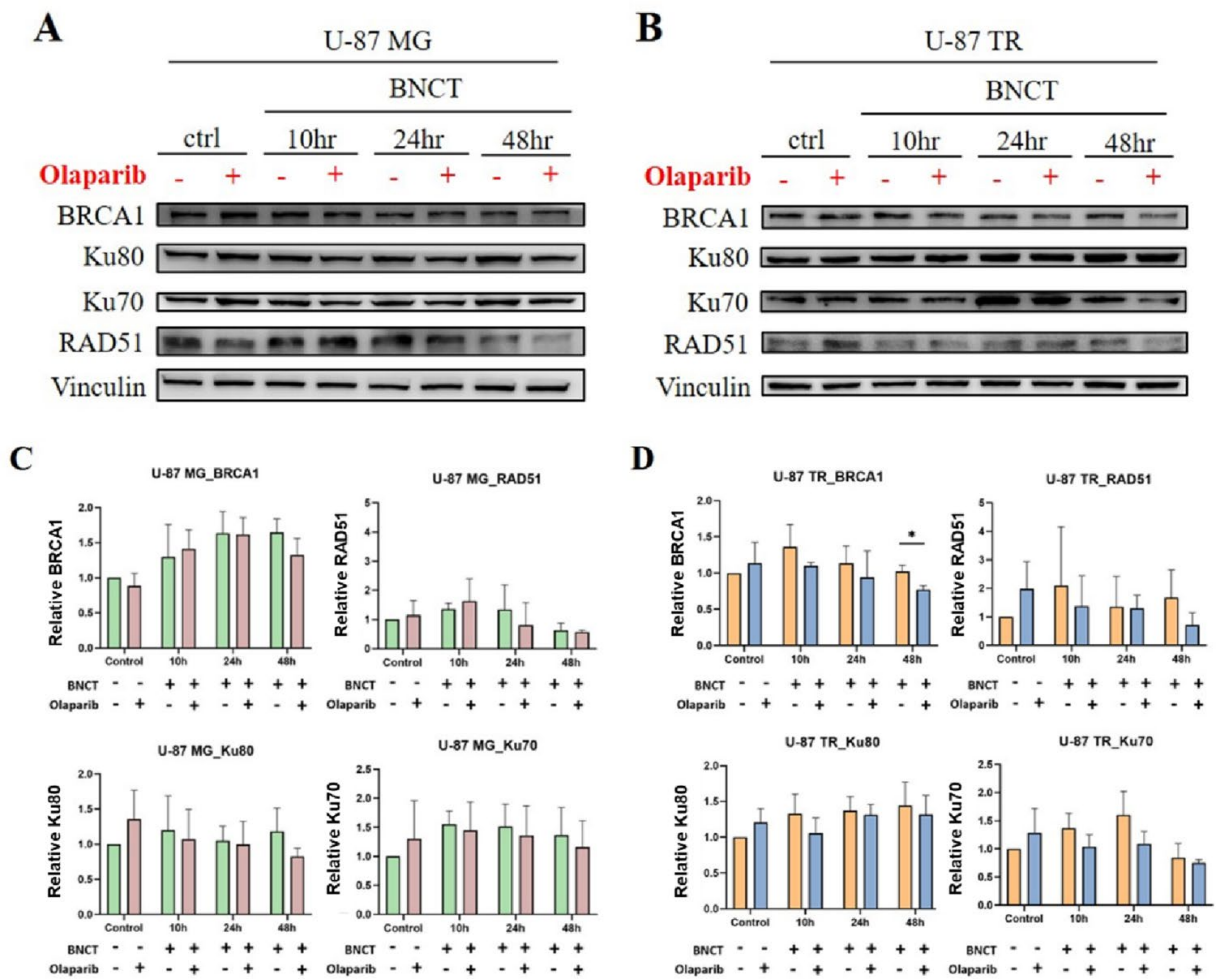
Homologous recombination and non-homologous end joining protein expression after treatment. Representative western blot analysis of HR and NHEJ repair proteins in U-87 MG (**A**) and TMZ-resistant U-87 TR (**B**) cells at 10, 24, and 48 h post-treatment. Vinculin served as a loading control. **C** and **D** Densitometric quantification of relative protein expression levels normalized to loading control. Data are presented as mean ± SD from three independent experiments. Statistical comparisons were performed using an unpaired, two-tailed Student’s *t*-test (*p < 0.05)

Given the sustained DNA damage observed in U-87 TR cells following combination treatment, we hypothesized that olaparib may interfere with DNA repair processes, amplifying the cytotoxic effects of BNCT [30]. To investigate this, we performed Western blot analysis to examine the expression of key HR mediators (BRCA1 and RAD51) and NHEJ mediators (Ku70 and Ku80) (Fig. 5).

In U-87 MG cells, BRCA1 expression was slightly elevated at all assay time points post-BNCT monotherapy (10, 24, and 48 h), and a similar trend was observed in the combination group, indicating minimal impact of olaparib on HR signaling in this cell line. RAD51 expression in U-87 MG increased at 10 h but declined later in both treatment groups (Fig. 5A; quantification shown in Fig. 5C).

Notably, U-87 TR cells showed a more dynamic HR response. BRCA1 expression initially rose after BNCT but was consistently suppressed by co-treatment with olaparib (Fig. 5B; quantification shown in Fig. 5D). Similarly, RAD51 levels were elevated by BNCT but reduced upon addition of olaparib, especially at 48 h post-irradiation. In contrast, expression levels of NHEJ-associated proteins Ku70 and Ku80 remained essentially unchanged in both cell lines and across treatment conditions, suggesting a limited role for NHEJ in mediating the observed effects. Overall, these results suggest that olaparib may affect BNCT-induced upregulation of the HR repair proteins, particularly in chemoresistant U-87 TR cells, thereby enhancing the therapeutic efficacy of BNCT.

### Distinct apoptotic mechanisms induced by combined olaparib and BNCT in U-87 MG and U-87 TR cells

**Fig. 6:**
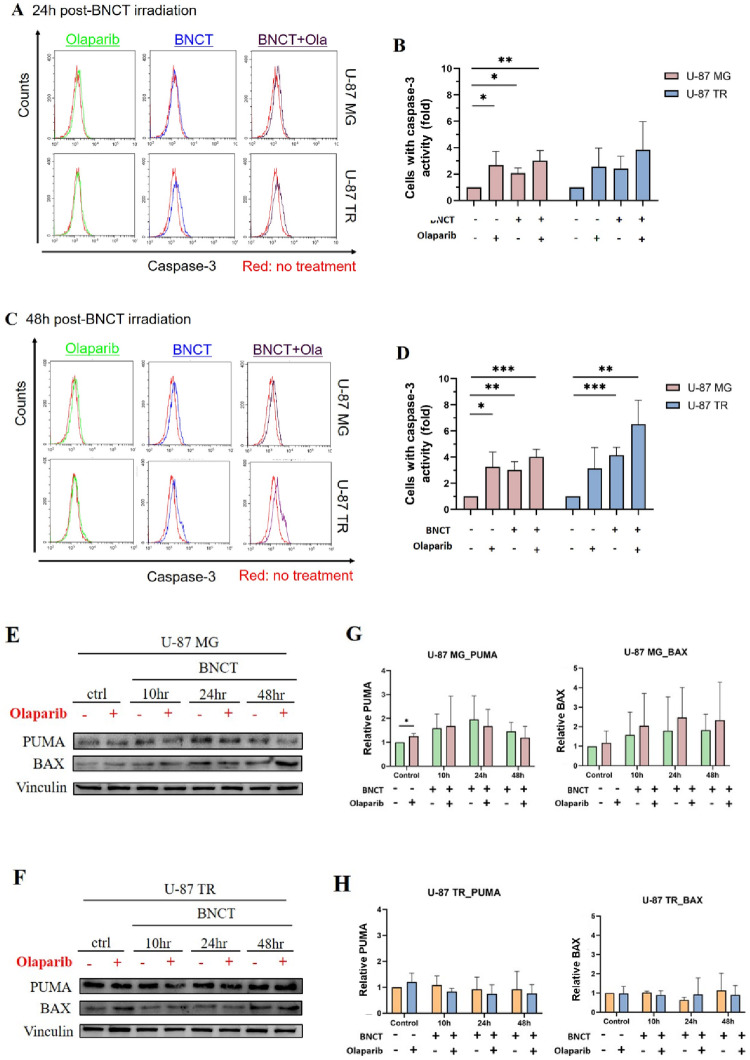
Caspase-3 activation and apoptotic protein expression following combined treatment. Representative flow cytometry histograms showing the apoptotic marker caspase-3 activity in U-87 MG and TMZ-resistant U-87 TR cells at 24 h (**A**) and 48 h (**C**) post-treatment. Caspase-3 activation was detected using PE-conjugated antibody staining; the red overlay represents the untreated control. **B** and **D** Quantification of caspase-3 activity is presented as a fold change in the percentage of positive cells relative to the control. **E** and **F** Representative western blot images of PUMA and BAX at 10, 24, and 48 h post-treatment. Vinculin served as the loading control. **G** and **H** Densitometric quantification of PUMA and BAX expression levels normalized to the loading control. Data are expressed as mean ± SD from three independent experiments. Statistical significance was determined using unpaired, two-tailed Student’s *t*-test comparisons (*p < 0.05, **p < 0.01, ***p < 0.001)

To investigate whether prolonged G_2_/M arrest contributes to apoptosis, we assessed caspase-3 activation in U-87 MG and U-87 TR cells at 24 and 48 h post-treatment (Fig. 6A–D). Combination treatment with olaparib and BNCT significantly enhanced apoptosis compared to BNCT alone, particularly in U-87 TR cells. At 24 h, caspase-3 activity increased to 3.846 ± 2.135-fold in U-87 TR cells (vs. 2.418 ± 0.952 with BNCT alone) and remained elevated at 48 h (6.517 ± 1.834 vs. 4.153 ± 0.602). In contrast, U-87 MG cells exhibited a more modest increase. Such heightened apoptotic sensitivity in U-87 TR cells likely reflects accumulated DNA damage and impaired repair capacity.

To elucidate underlying apoptotic pathways, we analyzed the expression of pro-apoptotic proteins PUMA and BAX (Fig. 6E–H). In U-87 MG cells, both treatments induced PUMA and BAX expression, with higher levels in the combination group. In contrast, U-87 TR cells showed no significant PUMA or BAX induction, suggesting apoptosis may proceed through PUMA-independent mechanisms.

These findings demonstrate that while the combination treatment enhances apoptosis in both cell lines, the underlying mechanisms differ: U-87 MG cells undergo apoptosis at least in part via a PUMA-mediated pathway, whereas U-87 TR cells likely engage alternative, non-canonical apoptotic pathways.

## Discussion

This study demonstrates that the PARP inhibitor olaparib significantly potentiates BPA-mediated BNCT, with the greatest effect in chemoresistant U-87 TR cells. The combination enhanced cytotoxicity through three converging mechanisms: sustained DNA double-strand break signaling, prolonged G_2_/M checkpoint arrest, and suppression of homologous recombination repair proteins BRCA1 and RAD51. These molecular effects culminated in increased apoptosis, proceeding via PUMA–BAX activation in U-87 MG cells and through PUMA-independent pathways in U-87 TR cells.

To further interpret these differential responses, both parental and TMZ-resistant cell lines were employed as a relevant model to evaluate the BNCT-sensitizing potential of olaparib under distinct DNA repair backgrounds. Although U-87 TR cells exhibited lower boron uptake than U-87 MG cells, BNCT in this study was performed under fixed irradiation conditions rather than adjusted for boron-dependent absorbed dose. Importantly, despite their lower boron levels, U-87 TR cells demonstrated stronger BNCT-induced DNA damage responses, including more persistent γH2AX signaling, greater suppression of BRCA1 and RAD51, prolonged G₂/M arrest, and distinct caspase-3 activation. These effects contrast with expectations if boron concentration were the primary determinant of BNCT efficacy, as reduced boron uptake would typically result in diminished DNA damage and weaker checkpoint activation. Therefore, the observed phenotype supports the conclusion that intrinsic, resistance-associated alterations in DNA damage repair, rather than boron concentration alone, are the major drivers of the differential BNCT responses between the two cell lines.

Our findings support a model in which BNCT imposes a high burden of complex DNA damage, and concurrent PARP inhibition prevents repair of both single-strand breaks and replication-associated double-strand breaks. In chemoresistant GBM, this dual disruption overwhelms the DNA damage response, maintaining checkpoint activation and directing cells toward apoptosis. The cell line-specific apoptotic signatures observed here suggest that BNCT–PARPi efficacy may depend on intrinsic differences or downstream apoptotic effectors.

These findings align with extensive evidence supporting PARP inhibitors as radiosensitizers [20, 29]. Olaparib has shown favorable pharmacokinetics and brain distribution in the Phase I OPARATIC trial (NCT01390571), supporting its clinical applicability for TMZ-resistant GBM [26]. Several other PARP inhibitors, including talazoparib, niraparib, and veliparib, are under active clinical investigation for their radiosensitizing potential [22, 39], expanding the potential drug repertoire for integration with BNCT. In summary, the tumor-selective cytotoxicity of BNCT and the established clinical profile of olaparib offer strong near-term translational potential for clinical application.

Our mechanistic data also highlights additional DNA repair targets that may potentiate BNCT efficacy. RAD51 inhibition markedly sensitized U-87 TR cells to BNCT (Supplementary Fig. 5), underscoring HR suppression as a viable strategy in resistant GBM. While RAD51 inhibitors are not yet clinically approved, next-generation compounds with improved BBB penetration could serve as either alternatives or complements to PARP inhibitors. Similarly, inhibitors of ATM (NCT03423628), DNA-PK (NCT02977780), and WEE1 (NCT01849146) are in clinical evaluation and could be strategically combined with BNCT to extend its applicability to specific patient subgroups, particularly those with repair-proficient or checkpoint-dependent tumors.

Checkpoint regulation emerged from this study as another actionable therapeutic target. Sustained phosphorylation of CDK1 at Y15 in U-87 TR cells indicates prolonged G₂/M arrest in response to unresolved DNA damage. While this arrest can facilitate repair, it may also prime the cells for apoptosis if damage persists [37]. Pharmacologic abrogation of this checkpoint, using WEE1 or CHK1/2 inhibitors, could prematurely drive damaged cells into mitosis, triggering mitotic catastrophe. Based on our time-course data, the 10–24-h post-irradiation window may represent an optimal period for introducing checkpoint inhibitors in combination regimens with BNCT.

Several limitations should be acknowledged. First, our results were derived from in vitro models, which lack the complexity of the in vivo tumor microenvironment and BBB dynamics. Second, although the U-87 MG and U-87 TR cell lines are useful for modeling TMZ sensitivity and resistance, they do not capture the full molecular and phenotypic heterogeneity of GBM. It remains unclear whether similar responses would be observed in vivo. Third, our analysis focused on a selected panel of DDR and apoptotic markers; broader molecular profiling, such as transcriptomics or proteomics, could reveal additional therapeutic targets or resistance pathways. Future work should include orthotopic animal models, expanded molecular profiling, and biomarker validation to identify patient subgroups most likely to benefit from BNCT–PARPi.

Despite these constraints, the use of an FDA-approved drug, olaparib, strengthens the translational value of our findings. Future work should investigate survival outcomes, BBB permeability, and long-term efficacy in clinically relevant, chemoresistant GBM animal models. Moreover, the identification and validation of predictive biomarkers, such as BRCA1, RAD51, MGMT methylation, or p53 mutation status, may enable patient stratification and personalized regimen design.

In conclusion, our findings establish the combination of olaparib and BNCT as a mechanistically grounded therapeutic strategy for chemoresistant GBM, characterized by disruption of DNA repair, sustained checkpoint activation, and induction of apoptosis. These mechanistic insights provide a strong rationale for translating BNCT–PARP inhibition into precision treatment strategies for TMZ-resistant GBM.

## Supplementary Information

Below is the link to the electronic supplementary material.

Supplementary Material 1

## Data Availability

The data supporting this study’s findings are not openly available due to reasons of sensitivity and are available from the corresponding author upon reasonable request. Data are located in controlled-access data storage at National Tsing Hua University.

## Abbreviations

7-AAD: 7-Amino-actinomycin D
ATM: Ataxia telangiectasia mutated protein
BAX: BA: Bcl-2-associated X protein
BCA: Bicinchoninic acid
BER: Base excision repair
BNCT: Boron neutron capture therapy
BRCA1: Breast cancer 1
BRCA2: Breast cancer 2
BSA: Bovine serum albumin
CDK1: Cyclin-dependent kinase 1
CHK2: Checkpoint kinase2
DDR: DNA damage response
DNA-PK: DNA-dependent protein kinase
DSBs: DNA double-strand breaks
FDA: Food and Drug Administration
GBM: Glioblastoma
Gy: Gray
γH2AX: Phosphorylated-H2AX
HR: Homologous recombination
HRP: Horseradish peroxidase
IC_50_: 50% growth-inhibitory concentration
NHEJ: Non-homologous end joining
PARP: Poly(ADP-ribose) polymerase
pCDK1: Phosphorylated-cyclin-dependent kinase 1
pCHK2: Phosphorylated-checkpoint kinase2
PE: Phycoerythrin
PFA: Paraformaldehyde
PUMA: p53 upregulated modulator of apoptosis
RER: Radiation enhancement ratio
SSBs: Single-strand breaks
THOR: Tsing Hua open-pool reactor
TMZ: Temozolomide
