# Supplementary material for: PARP Inhibition potentiates boron neutron capture therapy in chemoresistant glioblastoma via DNA repair disruption
Source: Jpn J Radiol. 2025 Dec 24;44(5):949–62. doi: 10.1007/s11604-025-01929-9 (PMC13144210; doi:10.1007/s11604-025-01929-9)
Supplement: Supplementary file 1 — Supplementary Material 1 [file 11604_2025_1929_MOESM1_ESM.docx]

**Supplementary Tables**

##

|  | Dose rate (Gy/minute) | |
| --- | --- | --- |
|  | **U-87 MG** | **U-87 TR** |
| Neutron | 1.2×10^-2^ | 1.2×10^-2^ |
| Gamma | 2.4×10^-2^ | 2.4×10^-2^ |
| ^10^B(n,α)^7^Li | 5.88×10^-1^ | 4.5×10^-1^ |

## Supplementary Table 1. Physical Dose rates of BNCT irradiation for U-87 MG and U-87 TR glioblastoma cells.

This table summarizes the measured dose rates (in Gy/min) for each radiation component during BNCT exposure at the Tsing Hua Open-pool Reactor. The BNCT dose was expressed as the physical neutron dose (Gy) calculated from the neutron fluence and energy deposition, without applying RBE or CBE conversion. Neutron and gamma doses were constant across both cell lines. The dose contributed by the ¹⁰B(n,α)⁷Li reaction, representing the boron-specific cytotoxic effect, was higher in U-87 MG cells than in the TMZ-resistant U-87 TR cells, potentially reflecting differences in boron uptake efficiency or intracellular distribution of boronophenylalanine (BPA).

| **Primary Antibody** | | | |
| --- | --- | --- | --- |
| **Name** | **Host** | **Brand** | **Condition** |
| Anti-γH2AX (Ser139) | Rabbit | Cell Signaling Technology #9718 | 1:400 in 3% BSA/PBS (ICC) |
| Anti-CHK2 | Rabbit | Cell Signaling Technology #2662 | 1:1000 in 3% BSA/TBST  (Western) |
| Anti-pCHK2 (Thr68) | Rabbit | Cell Signaling Technology #2197 | 1:1000 in 3% BSA/TBST  (Western) |
| Anti-Cyclin B1 | Mouse | Cell Signaling Technology #4135 | 1:1000 in 3% BSA/TBST  (Western) |
| Anti-CDK1 | Mouse | Cell Signaling Technology #9116 | 1:1000 in 3% BSA/TBST  (Western) |
| Anti-pCDK1 (Thr161) | Rabbit | Cell Signaling Technology #9114 | 1:500 in 3% BSA/TBST  (Western) |
| Anti-pCDK1 (Tyr15) | Rabbit | Cell Signaling Technology #4539 | 1:1000 in 3% BSA/TBST  (Western) |
| Anti-KU80 | Rabbit | Cell Signaling Technology #2180 | 1:1000 in 3% BSA/TBST  (Western) |
| Anti-KU70 | Rabbit | Cell Signaling Technology #4588 | 1:1000 in 3% BSA/TBST  (Western) |
| Anti-BRCA1 | Rabbit | Cell Signaling Technology #9010 | 1:1000 in 3% BSA/TBST  (Western) |
| Anti-RAD51 | Rabbit | Cell Signaling Technology #8875 | 1:500 in 3% BSA/TBST  (Western) |
| Anti-PUMA | Rabbit | Cell Signaling Technology #4076 | 1:500 in 3% BSA/TBST  (Western) |
| Anti-BAX | Rabbit | Cell Signaling Technology #2772 | 1:500 in 3% BSA/TBST  (Western) |
| Anti-Vinculin | Mouse | Sigma-Aldrich V9131 | 1:1000 in 3% BSA/TBST  (Western) |
| **Secondary Antibody** | | | |
| **Name** | **Host** | **Brand** | **Condition** |
| Anti-rabbit IgG-HRP | Goat | Arigo ARG65350 | 1:5000 in 5% NFDM/TBST  (Western) |
| Anti-mouse IgG-HRP | Goat | Arigo ARG65351 | 1:5000 in 5% NFDM/TBST  (Western) |
| Anti-mouse  IgG-Dylight 488 | Goat | Jackson | 1:400 in 3% BSA/PBS (ICC) |

## Supplementary Table 2. List of antibodies used in Western blot and Immunocytochemistry assays.


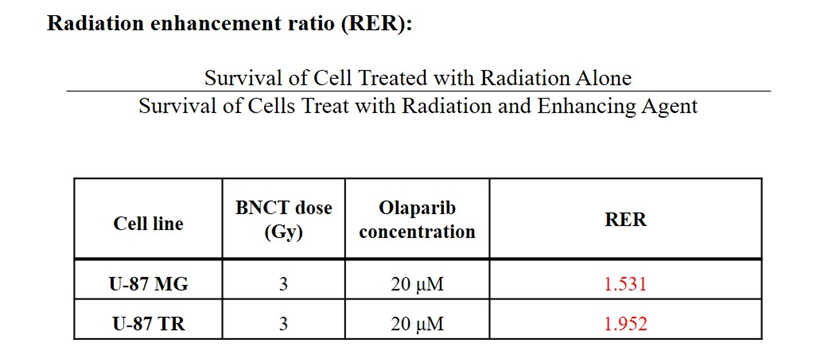


| **Cell line** | **BNCT dose (Gy)** | **Olaparib concentration** | **RER** |
| --- | --- | --- | --- |
| **U-87 MG** | 3 | 20 μM | 1.531 ± 0.450 |
| **U-87 TR** | 3 | 20 μM | 1.952 ± 0.774 |

## Supplementary Table 3. Olaparib functions as a potential radiosensitizer in BNCT.

RER was calculated for each independent experiment using the surviving fractions of BNCT alone and BNCT + olaparib, and the final RER value shown here represents the mean ± SD of three independent experiments. An RER value greater than 1 indicates radiosensitization. Olaparib enhanced the cytotoxic effect of BNCT in both cell lines, with a more substantial impact observed in the resistant U-87 TR cells (RER = 1.952 ± 0.774) compared to U-87 MG (RER = 1.531 ± 0.450), highlighting its potential to improve therapeutic efficacy.


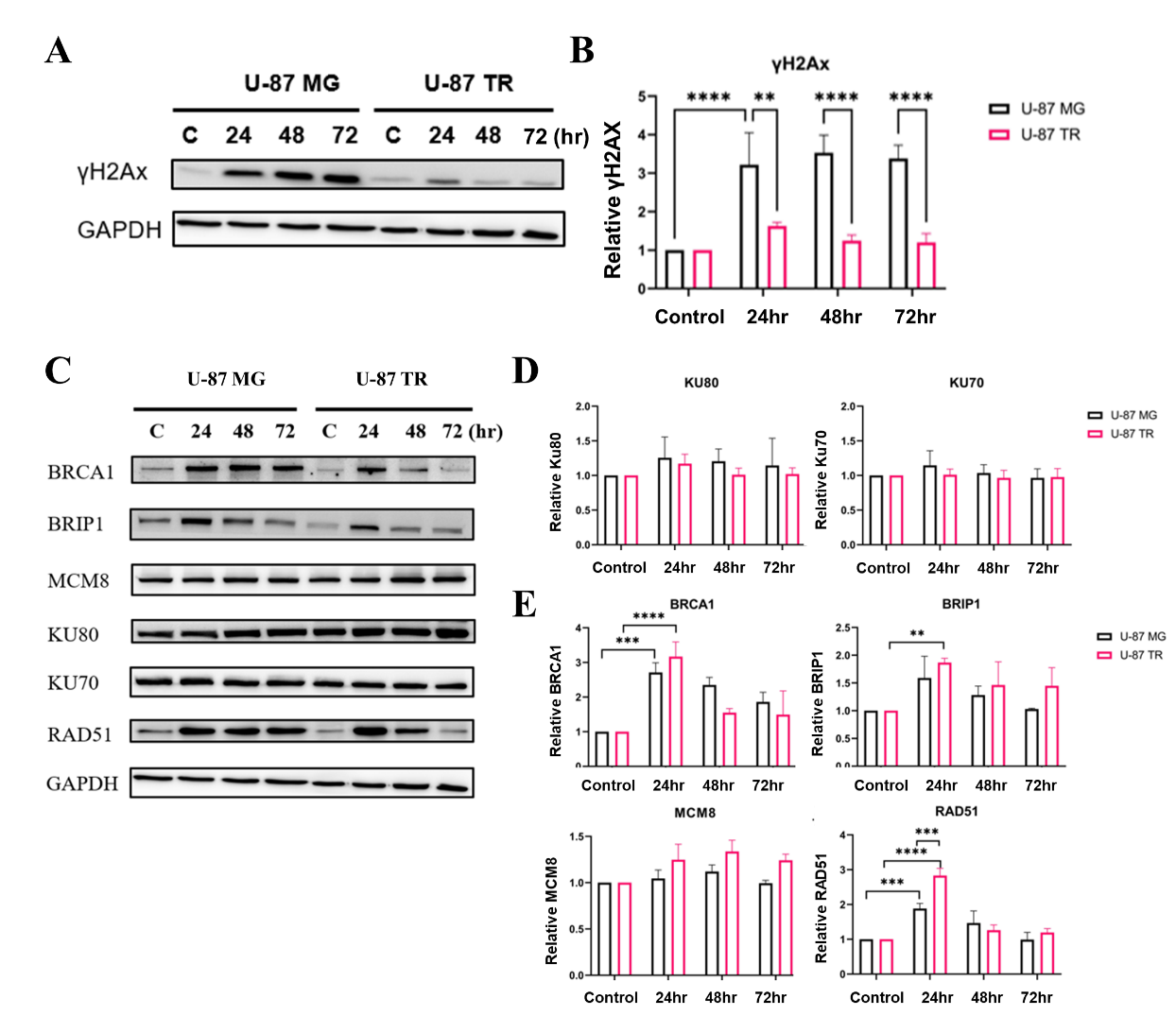
**Supplementary Figures**

**Supplementary Figure 1. Enhanced Homologous Recombination Confers TMZ Resistance in U-87 TR Cells.**

(A) Western blot analysis of γH2AX expression at the indicated time points after TMZ treatment in U-87 MG and U-87 TR cells. (B) Quantification of γH2AX expression levels was done with Image J. (C) Western blot analysis of NHEJ- and HR-related proteins. (D, E) Quantification of NHEJ- and HR-related protein expression. Data are expressed as mean ± SD from three independent experiments. Statistical significance was determined using unpaired, two-tailed Student’s *t*-test comparisons (**p* < 0.05, ***p* < 0.01, ****p* < 0.001, *****p* < 0.0001).


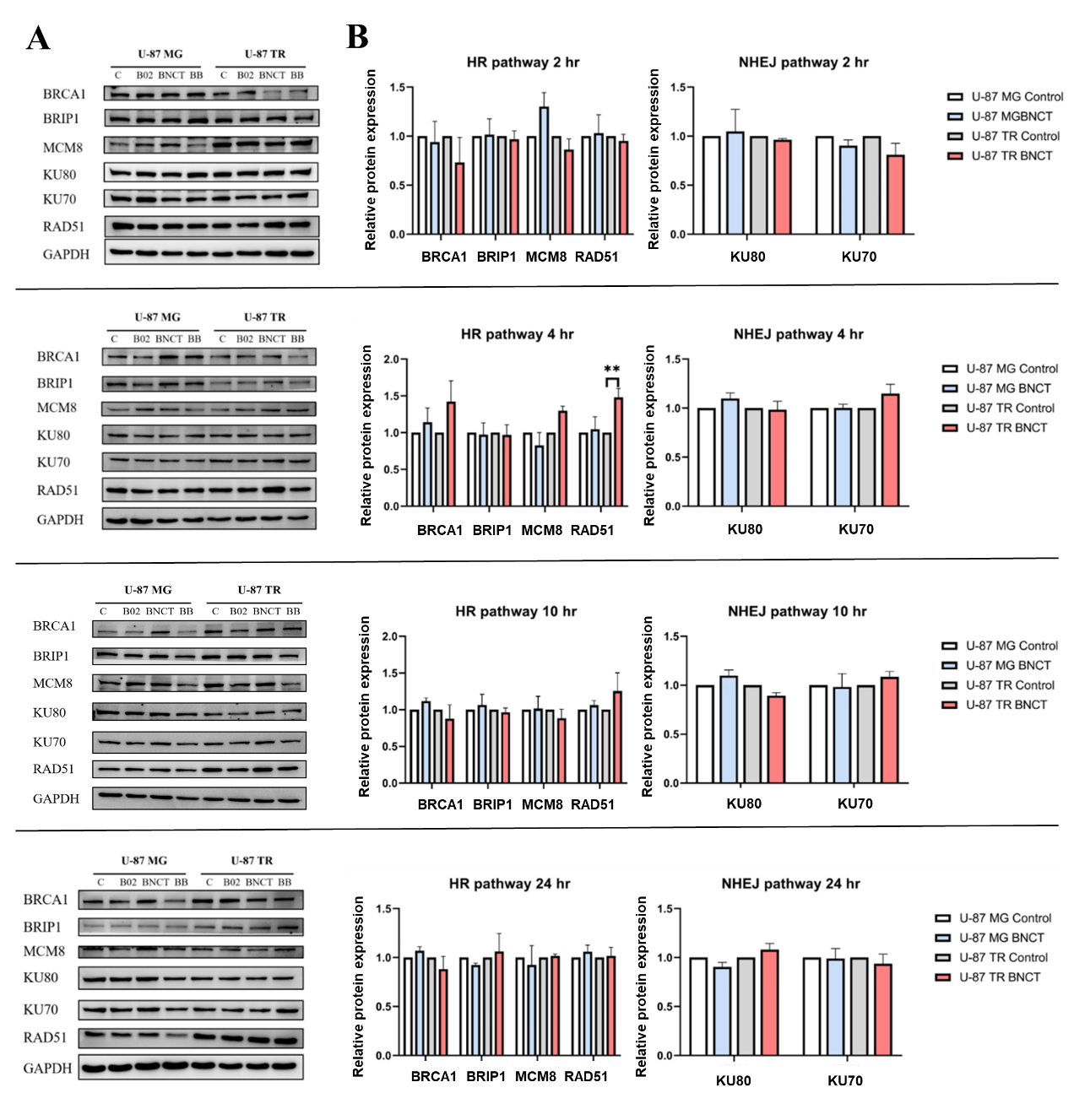


**Supplementary Figure 2. Rapid HR Pathway Activation Following BNCT in U-87 TR Cells.**

(A) Western blot analysis of HR- and NHEJ-related protein expression at 2, 4, 10, and 24 h post-BNCT irradiation in U-87 MG and U-87 TR cells. (B) Quantification of protein expression levels. Data are expressed as mean ± SD from three independent experiments. Statistical significance was determined using unpaired, two-tailed Student’s *t*-test comparisons (***p* < 0.01).

**
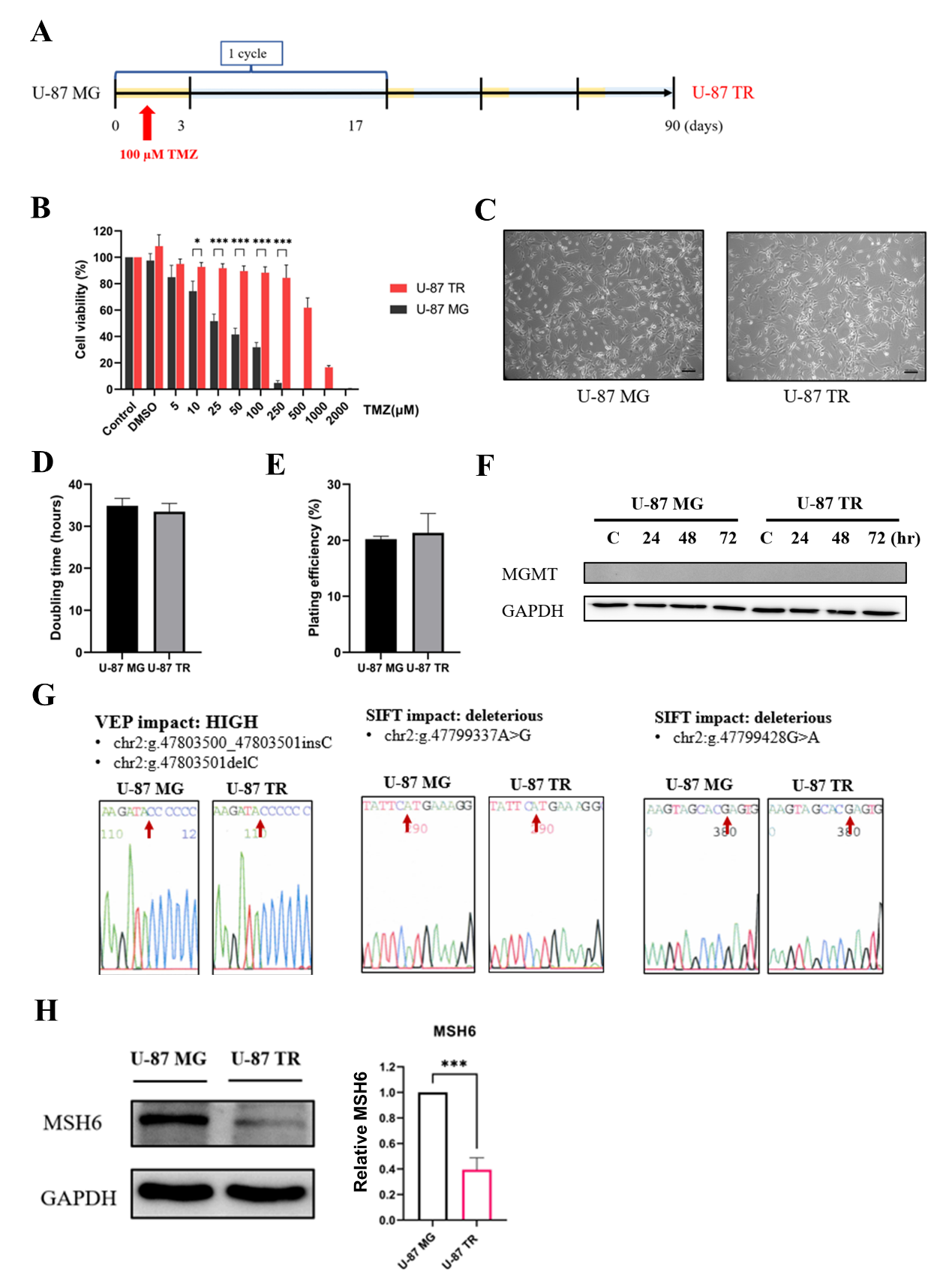
**

**Supplementary Figure 3.** **Characterization of TMZ-Resistant U-87 TR Cells.**

(A) Protocol for establishing TMZ-resistant U-87 TR cells. (B) CCK-8 assay showing TMZ IC₅₀ values for U-87 MG (32.71 μM) and U-87 TR (561.60 μM). (C) Representative phase-contrast images of U-87 MG and U-87 TR cell morphology. (D) Cells were collected at 24, 48, and 72 hours, and the doubling time of each cell line was calculated. (E) 300 cells were seeded and cultured in 6-well plates. The attached cells grew into colonies containing more than 20 cells. The plating efficiency of each cell line was calculated. (F) Western blot analysis of MGMT protein expression after TMZ treatment. (G) DNA sequencing comparison of four TCGA-reported MSH6 mutation sites between U-87 MG and U-87 TR; no mutations were detected in the resistant line. (H) Western blot analysis and quantification of MSH6 protein expression following TMZ treatment. Data are expressed as mean ± SD from three independent experiments. Statistical significance was determined using unpaired, two-tailed Student’s *t*-test comparisons (**p* < 0.05, ****p* < 0.001).


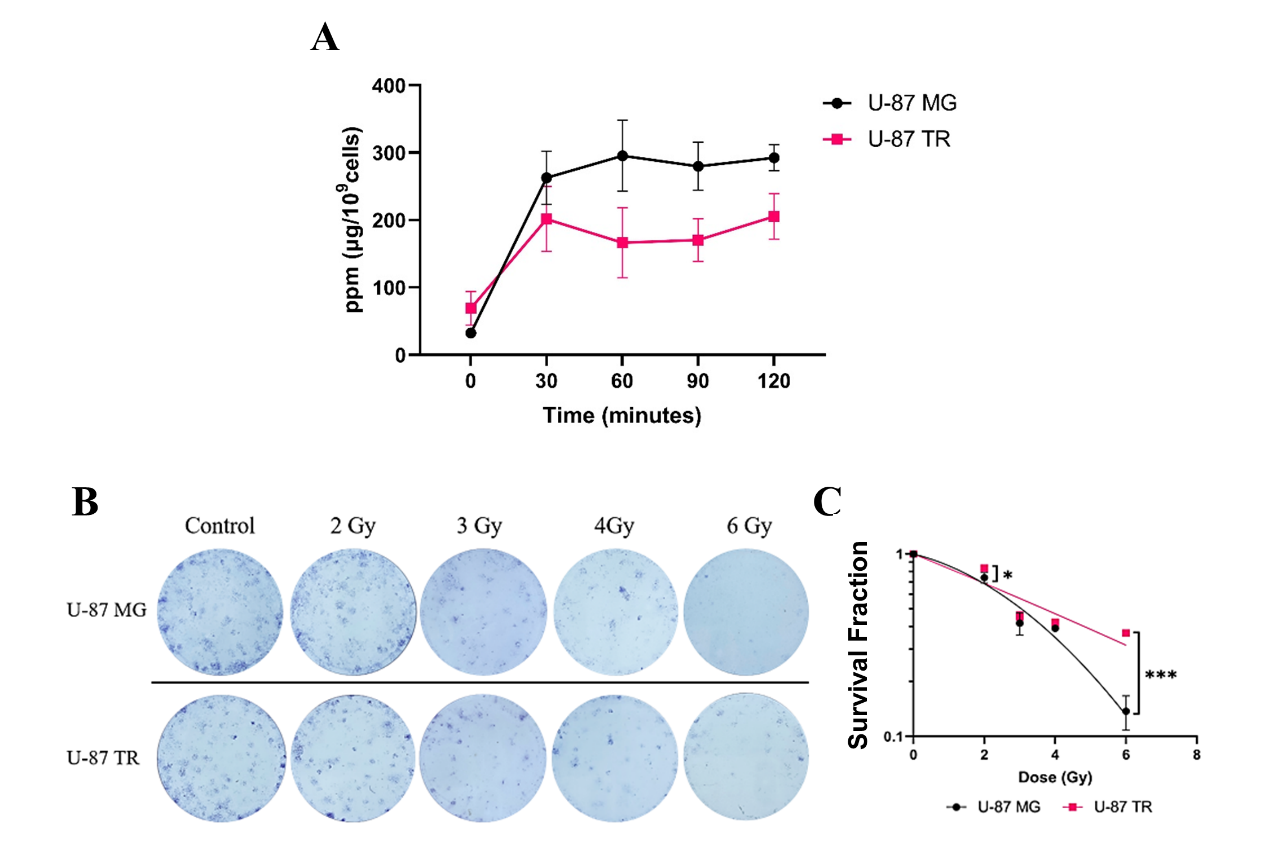


**Supplementary Figure 4. Boron Uptake and BNCT Dose–Response in U-87 MG and U-87 TR Cells.**

(A) Boron concentration in U-87 MG and U-87 TR cells at indicated time points after exposure to 25 μg ¹⁰B/ml BPA, measured by inductively coupled plasma–atomic emission spectrometry (ICP-AES). (B) Representative images of colony formation following BNCT at 2, 3, 4, or 6 Gy. Colonies were stained with crystal violet, and surviving fractions were calculated. (C) Quantification of surviving fractions; U-87 TR cells exhibited higher survival than U-87 MG cells at 6 Gy. Data are expressed as mean ± SD from three independent experiments. Statistical significance was determined using unpaired, two-tailed Student’s *t*-test comparisons (**p* < 0.05, ****p* < 0.001).


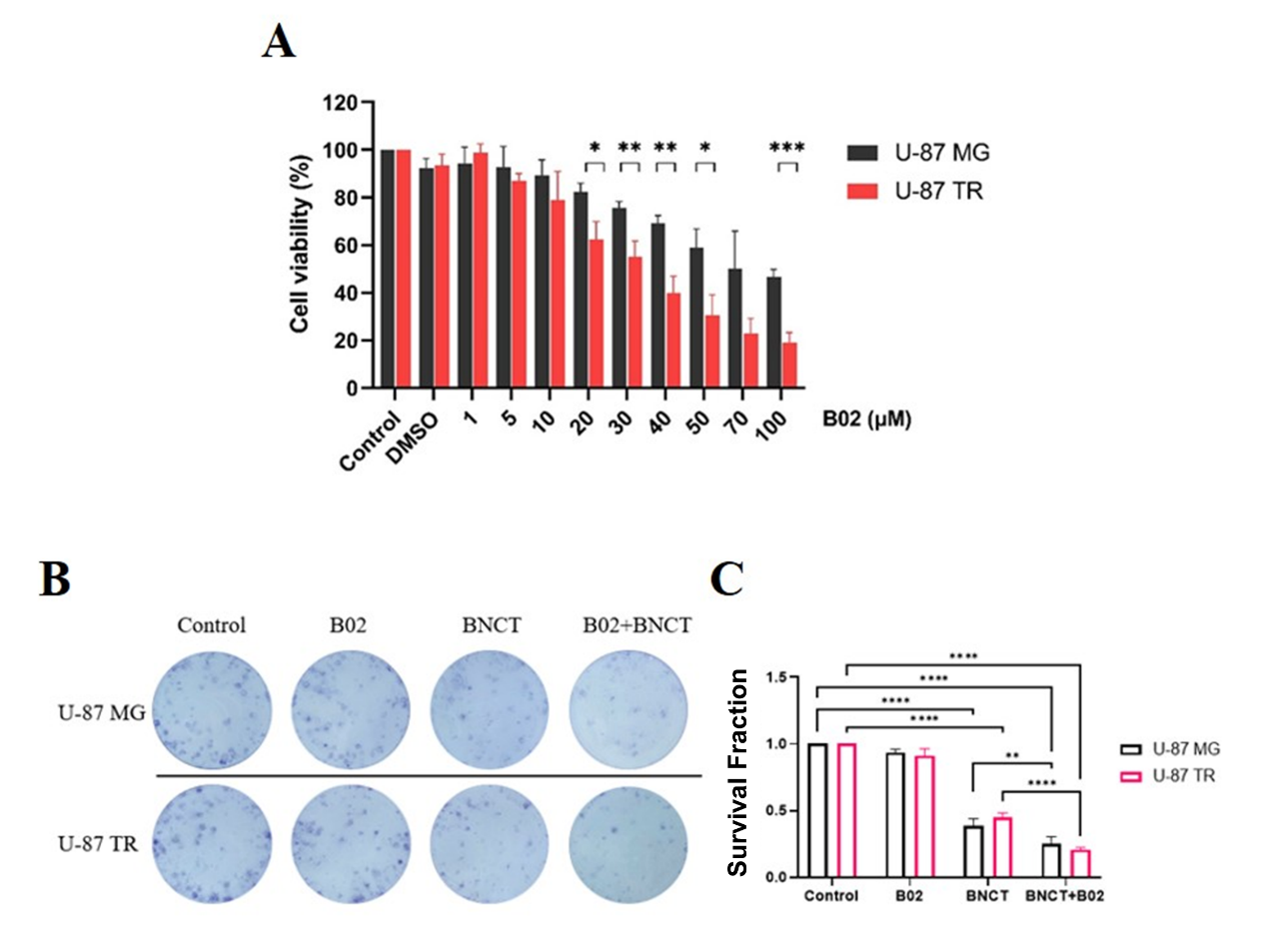


**Supplementary Figure 5. RAD51 Inhibition Enhances BNCT Sensitivity in U-87 TR Cells.**

(A) CCK-8 assay showing IC₅₀ values of the RAD51 inhibitor B02 in U-87 MG (42.29 μM) and U-87 TR (32.33 μM) cells. (B) Representative images of colony formation following treatment with B02 alone, BNCT alone (3 Gy), or the combination. Colonies were stained with crystal violet, and surviving fractions were calculated. (C) Quantification of surviving fractions shows a significant reduction with the combination treatment compared to BNCT alone. Data are expressed as mean ± SD from three independent experiments. Statistical significance was determined using unpaired, two-tailed Student’s *t*-test comparisons (**p* < 0.05, ***p* < 0.01, ****p* < 0.001, *****p* < 0.0001).
